# Comparison of In Vivo Intradiscal Pressure between Sitting and Standing in Human Lumbar Spine: A Systematic Review and Meta-Analysis

**DOI:** 10.3390/life12030457

**Published:** 2022-03-20

**Authors:** Jia-Qi Li, Wai-Hang Kwong, Yuk-Lam Chan, Masato Kawabata

**Affiliations:** 1 Department of Rehabilitation Sciences, Hong Kong Polytechnic University, Hong Kong, China; jiajiaqi.li@connect.polyu.hk (J.-Q.L.); 21121079g@connect.polyu.hk (Y.-L.C.); 2 Physical Education & Sports Science Academic Group, National Institute of Education, Nanyang Technological University, Singapore 637616, Singapore; masato.kawabata@nie.edu.sg

**Keywords:** low back pain, intradiscal pressure, in vivo measure, posture

## Abstract

Background: Non-specific low back pain (LBP) is highly prevalent today. Disc degeneration could be one of the causes of non-specific LBP, and increased intradiscal pressure (IDP) can potentially induce disc degeneration. The differences in vivo IDP in sitting and standing postures have been studied, but inconsistent results have been reported. The primary objective of this systematic review is to compare the differences in vivo IDP between sitting and standing postures. The secondary objective of this review is to compare effect size estimates between (1) dated and more recent studies and (2) healthy and degenerated intervertebral discs. Methods: An exhaustive search of six electronic databases for studies published before November 2021 was conducted. Articles measuring in vivo IDP in sitting and standing postures were included. Two independent researchers conducted the screening and data extraction. Results: Ten studies that met the inclusion criteria were included in the systematic review, and seven studies with nine independent groups were included in meta-analyses. The sitting posture induces a significantly higher IDP on the lumbar spine (SMD: 0.87; 95% CI = [0.33, 1.41]) than the standing posture. In studies published after 1990 and subjects with degenerated discs, there are no differences in vivo IDP between both postures. Conclusions: Sitting causes higher loads on the lumbar spine than standing in the normal discs, but recent studies do not support this conclusion. Furthermore, the degenerated discs showed no difference in IDP in both postures.

## 1. Introduction

Low back pain (LBP) has become the leading cause of disabilities and absenteeism worldwide [1,2]. A study in 2018 showed that more than 500 million people globally suffer from this symptom and are affected by concurrent comorbidities such as depression, diabetes, and other musculoskeletal disorders [3,4]. These problems, when chronic, put heavy economic and psychological burdens on patients. For example, in the US, approximately $784 million was spent on surgery and $1.8 billion on conservative treatments in 12-month care in 2018–2019 [5,6,7].

Intradiscal pressure (IDP) is the hydrostatic pressure measured in the nucleus pulposus of the intervertebral disc (IVD). As the innervated structure of the IVD [8,9], it is recognized as one of the potential causes of LBP. Studies have discovered that an increased IDP may accelerate the process of disc degeneration [10,11,12]. In the degenerated disc, the amount of incompressible fluid decreases, and the nucleus pulposus cannot maintain even pressure on the adjacent annulus fibrosus and endplates, which could be a mechanical cause of LBP [13]. Thus, understanding the factors that could affect the IDP could help clinicians and scientists to develop and modify the strategy for managing LBP.

Many studies have investigated the relationships between lumbar IDP and postures. Although finite models have calculated IDP in recent years, in vivo measurements tend to show more accurate data. A previous study suggests that a finite model is never “valid” for all situations and applications; instead, validation is tied to a specific topic of interest [14]. Moreover, a number of these validation studies may fail in providing complete modelling methodologies and validation data [15]. In the 1960s, Nachemson et al. implanted a pressure transducer into the IVD to measure the IDP directly [16]. In earlier studies, they reported that sitting increases the IDP by 40% more than standing [17]. In 1975, Andersson et al. performed a similar experiment using a subminiature pressure transducer and found that the IDP in standing is about 35% of that in relaxed sitting without back support [18]. Since then, sitting has been considered a risk factor that induces high lumbar IDP.

With the continuous advancement in transducer design, results of the in vivo IDP measures do not coincide with those of previous studies [19]. More recently, new methods such as internal spinal fixators and vertebral body replacement (VBR) have been seen as alternatives to obtaining in vivo IDP measurements. Similarly, results from current IDP studies are not consistent with earlier results and do not support the hypothesis that sitting increases the load on the lumbar spine. For example, a study that estimated IDP by measuring disc heights inferred that standing could impose higher loads on the lumbar spine than sitting [20]. Based on more recent studies, the strategy of reducing sitting time to prevent LBP may not be valid. A systematic review in 2008 [21] evaluated the effect of posture on IDP data and concluded that sitting and standing have a similar level of IDP. In addition, a review in 2015 conducted several comparisons of different postures in vivo and vitro measurement, and their conclusion supports the similar IDP as well [22]. The limitation in measurement technology could be a confounding factor that affects the accuracy of the IDP measure. For example, the insertion of a needle transducer will result in abnormal muscle contraction when changing postures [23]. To date, no study has evaluated the effect of sitting and standing on IDP using a meta-analysis. Therefore, this study aims to estimate the effect size of lumbar IDP in vivo measurement in sitting compared to standing posture. This study will also compare the effect size estimate between (1) dated and more recent studies and (2) healthy and degenerated IVDs. 

## 2. Methods

### 2.1. Identification and Selection of Studies

**Figure 1:**
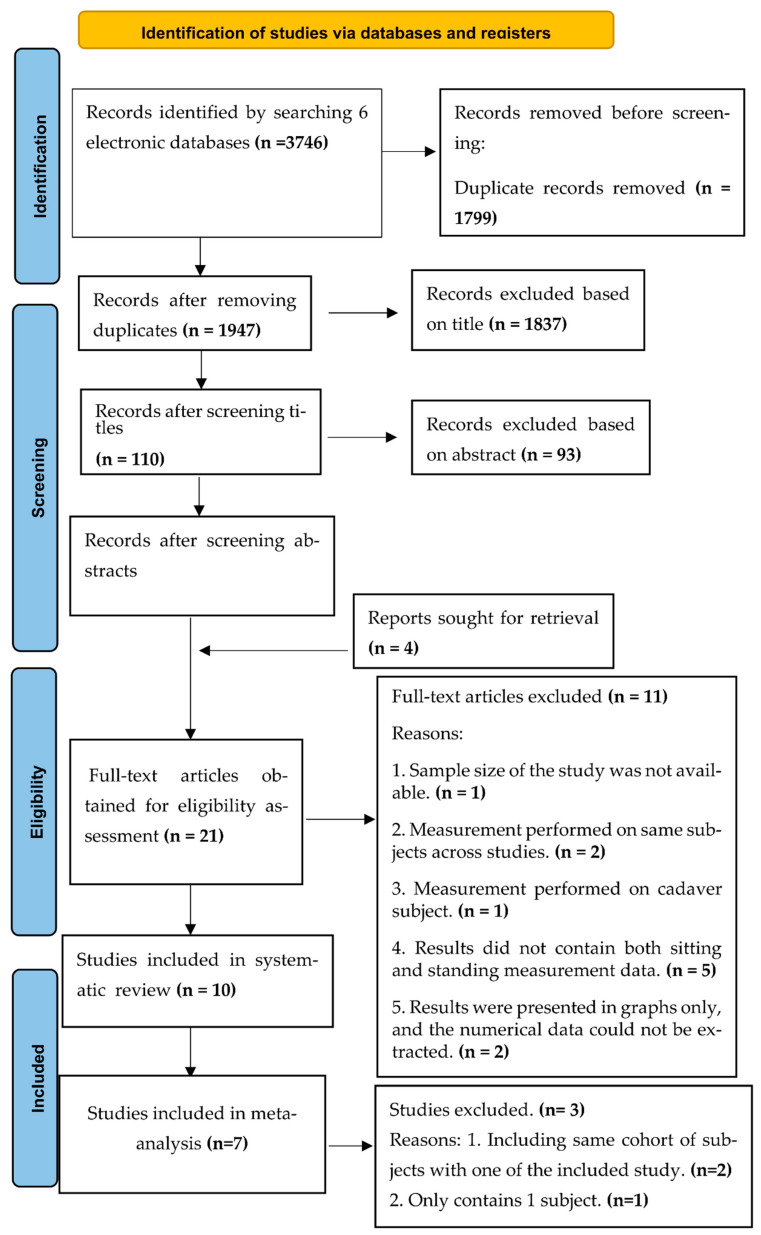
Flow diagram showing the selection of studies.

A systematic search was performed by two reviewers (Li J and Chan M) for articles published before November 2021, and there was no restriction on the earliest publication date. Six electronic databases, namely, Google Scholar, Scopus, PubMed, Web of Science, EMBASE, and Cochrane Library, were used to search for the related articles. The detailed searching strategy is presented in Appendix A. The keywords used in the literature search included healthy adults, sitting posture, standing posture, in vivo spinal loads, and in vivo IDP. Hand searching was also performed to obtain additional information by Li J and Chan M. The reference lists of the included studies were reviewed (backward tracking), and literature citing the included studies were tracked (forward tracking) to identify additional studies. For each paper, the ‘similar articles’ option in the PubMed database was used to further expand the search. Two reviewers (Li J and Chan M) screened for potentially eligible studies. Figure 1 illustrates the searching process. Disagreements regarding the eligibility of studies were resolved by discussion with a third reviewer (Kwong PWH).

Studies were included in the review if they (1) involved in vivo IDP measurement in both sitting and standing postures, (2) involved measurements with intervertebral body replacement, and (3) included spinal loading data of healthy adults. Studies were excluded if they (1) investigated in vitro measurement of IDP, (2) did not report the central tendency and/or variability of the outcome of interest, and (3) were letters to the editor, case studies, case series or review articles. For the relevant papers that did not provide sufficient data, we contacted the corresponding author to acquire the data. The article written in Japanese was reviewed by a researcher who is a native Japanese speaker ( Kawabata M).

### 2.2. Quality Assessment

Because all included studies were cross-sectional studies and no standard assessment tools were used to assess their quality, we referenced an approach developed by Friedemann et al. [24] that mainly considers five elements: (1) blinding of outcome assessment, (2) incomplete outcome data, (3) selective reporting, (4) precision of measurement methods, and (5) representative samples. Each of these outcomes is given a mark, resulting in a maximum of 5 marks for a cross-sectional study. We classified a paper as having moderate quality if it scored 3 or more marks. Studies with a quality score below 3 were considered low quality and were not included in the meta-analysis.

### 2.3. Data Extraction

Full-text reviews were performed on the selected articles after the title and abstract screening. Two reviewers (Li J and Chan M) extracted the data from articles independently. A standardized data extraction form was used to extract the data from the included studies. The extracted information included the sample size, characteristics of participants (age, gender, and disc condition), and outcomes (type of outcome measures and means and standard deviations (SDs) of the outcomes). For one study that measured the IDP at two spinal levels [25], we extracted the value of the L4-5 level only to ensure that individual data were not repeatedly included in the meta-analysis.

### 2.4. Data Synthesis and Analysis

The primary outcome measures were direct measures of the participants’ IDP or force at the lumbar level (containing the internal fixators with several segments, including adjacent thoracic vertebrae). Mean values and the SDs of the IDP were used to estimate the effect size. The mean value and SD were either directly extracted from the study or calculated manually according to the respective data of each subject provided in the article.

The statistical analyses were performed using the R language and the Meta package [26]. In addition, a trial sequential analysis was conducted to determine the required information size and adjust thresholds for significance [27]. The trial sequential analysis was conducted using the Trial Sequential Analysis software (The Copenhagen Trial Unit, Denmark). Where studies recruited the same batch of subjects, the most recent study was included in the meta-analysis. The study with only one subject was excluded from the analysis. As the relevant studies used different units of measurement, the standardized mean difference (SMD) was used to estimate the effect size.

### 2.5. Measuring Heterogeneity and Publication Bias

The I^2^ statistic was used to quantify statistical heterogeneity. A random-effects model would be used for each meta-analysis if the I^2^ statistic result were significant (*p* < 0.05) or if the I^2^ value were more than 50%, indicating significant heterogeneity. If the I^2^ statistic was non-significant, a fixed-effects model would be used, and a funnel plot was added to visualize the publication bias. 

The degree of publication bias was determined using a funnel plot and Egger’s regression test. In the funnel plot, the Hedges’ g of each study was plotted against its standard error. Egger’s regression test determines whether the intercept of the precision (inverse of the standard error) regression line of each study against the weighted effect size deviates significantly from 0. The funnel plot is skewed, according to a statistically significant Egger’s regression test. For Egger’s regression test, a statistical significance threshold of *p* < 0.1 was used.

### 2.6. Subgroup Meta-Analyses

Three subgroup analyses were conducted to determine whether the effect size estimate is affected by other factors. (1) An analysis of the differences between studies published before and after 1990. This cut-off point was selected since more advanced methods to measure the spine load was developed. (2) An analysis of the differences between normal and degenerated discs, and (3) an analysis of the differences in loading between L3-4 and L4-5 discs. All the statistical tests were two-tailed, and we set α = 0.05.

### 2.7. Meta-Regression

A meta-regression is used to quantify the relationship between the effect size of posture and the year of publication and total sample size of each study [28].

## 3. Results

### 3.1. Identification and Selection of Studies

The search identified 1947 records after duplicates were removed. Two reviewers reviewed these records. We excluded 1930 articles after the title and abstract screening. According to the inclusion and exclusion criteria, an additional four reports were identified and retrieved through backward and forward searches. Twenty-one relevant articles were identified, and a full-text review was conducted. Eleven articles were excluded after a full article review. Appendix B illustrates the excluded studies and the reasons for their exclusion.

Finally, ten cross-sectional studies were included in the systematic review after assessing their eligibility. These studies were published between 1964 and 2013 and comprised nine full-text articles published in English and one article in Japanese. The selected studies measured in vivo IDP or force during human movement and daily activities.

Three studies [29,30,31] comprised repetitive measures within the same cohort of subjects, and thus only the latest study from the study group [30] was included in the meta-analysis. One study was excluded from the meta-analysis since only one subject participated in the study. Of the remaining seven studies, 110 and 100 subjects’ data were included in the sitting and the standing groups, respectively.

### 3.2. Characteristics of Included Studies

Table 1 summarizes the characteristics of the included studies. Different intradiscal measurement methods were used. Measurements were taken at L3-L4 [25,32,33] and L4-L5 levels [25,32,33,34,35]. Four studies measured IDP between T12 and L5 levels [30,31,36]. Subjects included patients with LBP [25,32,33], healthy volunteers [34,35,37], and patients with vertebral fractures [29,30,31,36]. Three studies used a liquid-filled transducer [25,32,33], two studies used a piezoresistive pressure transducer [34,37], one study recorded telemetered internal spinal fixation devices [36] and three studies recorded vertebral body implant loading [29,30,31].

### 3.3. Quality of Studies

Table 2 shows the results of the quality assessment. Eight studies scored four marks [25,30,31,32,33,36,37,38], and only two studies scored three marks [34,35], which is defined as a moderate quality level. Of these two studies, one study conducted in 2001 [35] only involved one subject with a normal L4-5 disc and could not be analyzed in the meta-analysis; the other study conducted by Sato [34] in 1999 lacked the direct value of IDP in LBP patients. All of the studies lacked assessment blinding, which is restricted to the measurement procedure, and the measurement methods were described in detail. Data were acquired from multiple times; subjects were typical LBP patients [32,33], healthy volunteers [25,34,35,37] and patients with vertebral fractures [29,30,31,36]; the degree of disc degeneration was diagnosed by MRI; and postures were defined clearly in these studies.

### 3.4. IDP Difference between Sitting and Standing Postures

**Figure 2:**
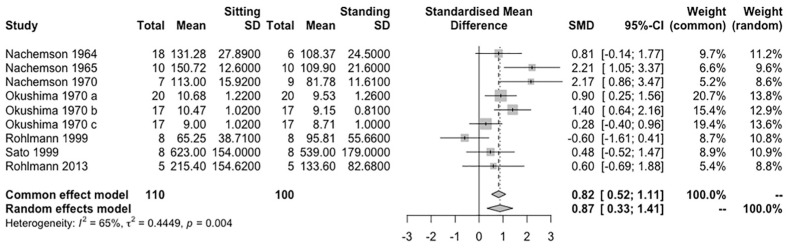
Effect of sitting compared to standing in IDP outcomes.

Seven studies with nine independent groups (involving 110 participants) were pooled in a meta-analysis. The R results showed that sitting induces a significantly higher IDP on the lumbar spine than standing (Hedges’ g = 0.87, 95% CI = 0.33–1.41, *p* < 0.01) and the effect is homogeneous (I^2^ = 65%, *p* = 0.04) (Figure 2).

SMD (95% CI) of the effect of sitting compared to standing on measures of IDP on the lumbar spine by pooling data from seven studies (n = 210). The sizes of the squares indicate the relative weight of each study

**Figure 3:**
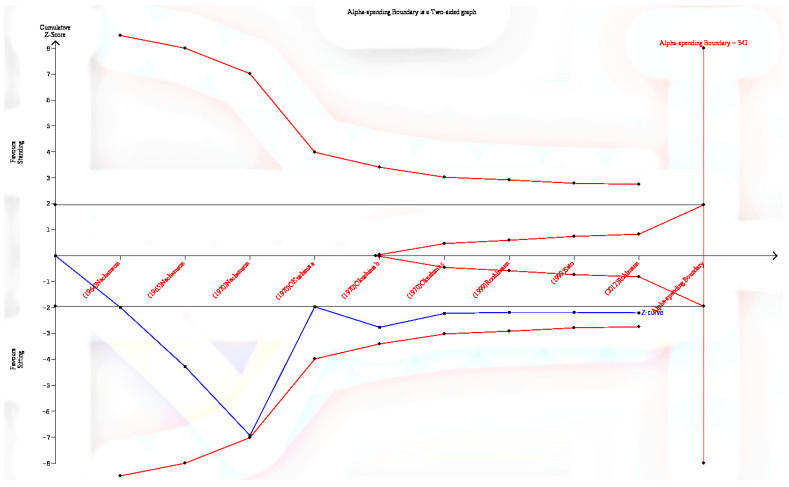
Results of the trial sequential analysis of the effect of sitting compared to standing in IDP outcomes.

In the trial sequential analysis (Figure 3), the last point of the Z-curve is outside the conventional test border, but inside the monitoring boundaries. As a result, while there is a statistical difference in the conventional meta-analysis, we cannot draw the same conclusion in trial sequential analysis. Besides, the analysis also indicates that the number of the study included in this review has not reached the estimated required information size. 

Nachemson and colleagues’ studies [32,33,37] were conducted in 1964, 1965, and 1970. The measurements in these studies were taken in normal discs and included two levels (L3-4 and L4-5). All results showed that sitting induces a higher load on the lumbar spine than standing, by more than 20–40% in values. In the sitting position, it is not shown whether the lower disc has a higher IDP. However, in the standing position, the lower disc tends to have a higher IDP.

Okushima’s study [25] in 1970 included subjects with different levels of disc segments (L3-4 and L4-5) and conditions. They conducted the experiment on 72 participants. Some participants received the measure in two spinal segments. Despite the disc condition (normal or pathological), the L4-5 disc has a higher IDP value in both postures. As the disc condition changes from normal to highly degenerated, the IDP difference between sitting and standing decreases from 1.32 to 0 (kg/cm^2^). This result showed that sitting does not always induce a higher IDP on the lumbar spine than standing.

Sato et al. [34] explored the IDP value at L4-5 in LBP patients and healthy volunteers. They measured IDP in the horizontal and vertical planes. For this review, we used the IDP in the vertical plane in the meta-analysis. The IDP values of LBP patients were displayed in histograms only. The results indicated that the degenerated disc tends to have a smaller IDP.

Rohlmann implanted internal spinal fixators to measure the in vivo IDP. In a study published in 1999 [36], all of the nine subjects were patients with degenerative, old or fresh vertebral fractures in the lumbar level. Before anterior interbody fusion surgery, the means of IDP in sitting and standing postures are 65.25 N and 95.81 N, respectively. After the surgery, these values increase to 94.39 N and 116.83 N, respectively. Standing results in a higher IDP than sitting. In 2001, Wilke et al. [35] used an implanted transducer to measure the IDP in one subject. The results showed a slightly higher IDP in standing than in sitting postures (0.5 to 0.46 MPa).

In studies conducted in 2008, 2010, and 2013 [29,30,31], Rohlmann et al. used telemeterized vertebral body replacement, which is developed by internal spinal fixators, and realized remote and multiple measurements. The three studies were conducted in repeated subjects; thus, the meta-analysis used the study of 2013. However, the length of time after surgery was different, from the first postoperative month to 6–12 months. Based on the results, the patients with longer recovery times tend to have a lower IDP. 

### 3.5. Publication Bias

**Figure 4:**
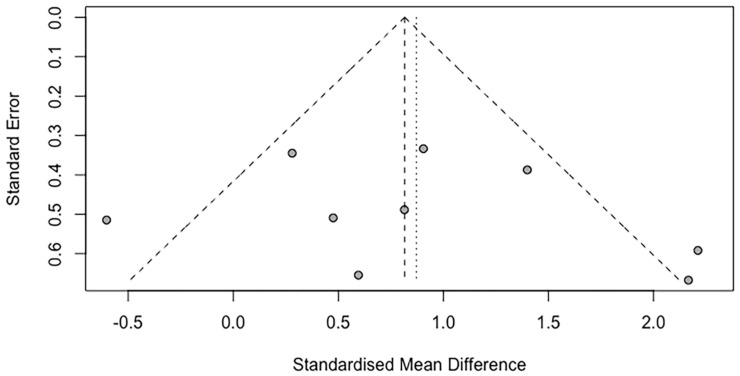
Funnel plot of the standard errors of each study against the standardized mean difference.

The funnel plot appeared symmetrical for the meta-analysis (Figure 4). According to Egger’s regression test, the intercept of the regression line did not substantially depart from 0 (intercept = 0.13; *p* = 0.54, 95% CI = −2.00, 2.68), indicating the absence of publication bias.

### 3.6. Sensitivity Analysis

**Figure 5:**
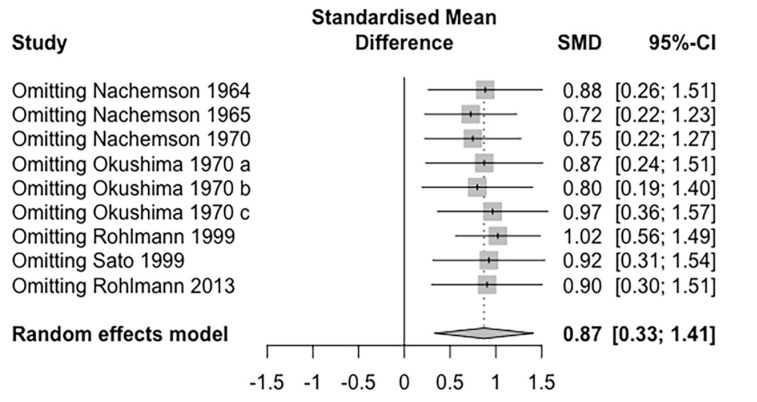
Leave-one-out sensitivity analysis of the impact of posture on IDP.

A leave-one-out meta-analysis was performed to evaluate the influence of each study on the overall effect size estimate. The seven studies were included in the leave-one-out analysis. The effect size estimate shrank, but it remained statistically significant (Hedges’ g = 0.87, 95% CI = 0.33–1.41) (Figure 5).

### 3.7. Subgroup Analyses

Three subgroup analyses were conducted based on the year of publication, the IVD condition, and disc levels.

**Figure 6:**
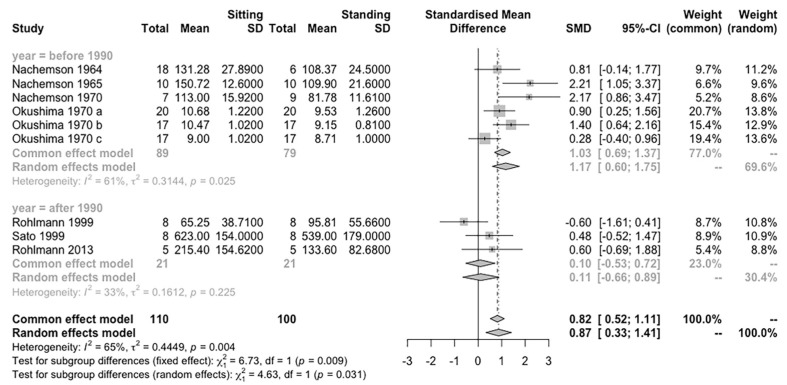
Subgroup analysis according to publication years for IDP difference between sitting and standing postures.

The test for subgroup differences in publication year suggested a statistically significant subgroup effect (*p* = 0.03), meaning that publication year significantly modifies the effect of IDP between sitting and standing postures. However, there is unexplained heterogeneity between the studies within each of the subgroups (studies after 1990: I^2^ = 33%; studies before 1990: I^2^ = 61%). Therefore, the validity of the differences effect estimate for each subgroup is uncertain, as individual trial results are inconsistent (Figure 6).

**Figure 7:**
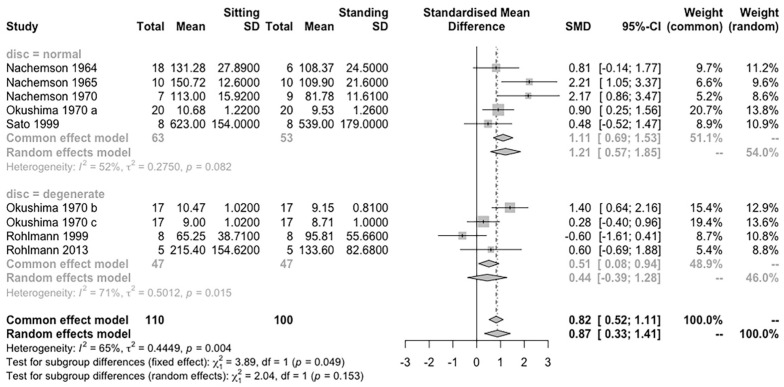
Subgroup analysis according to disc conditions for IDP difference between sitting and standing postures.

The test for subgroup differences in different disc conditions suggested that there is no statistically significant subgroup effect (*p* = 0.15). There is substantial unexplained heterogeneity between the studies within each of the subgroups (degenerated disc: I^2^ = 71%; normal disc: I^2^ = 52%). However, the effect of normal discs was significant (Hedges’ g = 1.21, 95% CI = 0.57–1.85). Therefore, the validity of the differences effect estimate should be interpreted cautiously (Figure 7).

**Figure 8:**
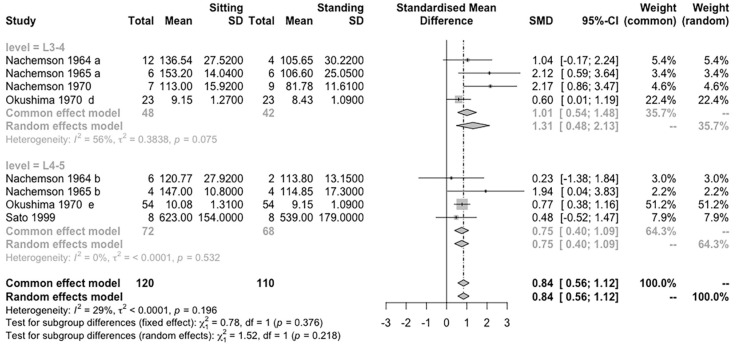
Subgroup analysis according to disc levels for IDP difference between sitting and standing postures.

The analysis for subgroup differences in different disc levels suggested that there is no statistically significant subgroup effect (*p* = 0.218) (Figure 8). The subgroup of L3-4 has heterogeneity between studies (I^2^ = 56%). The overall results show posture posed a comparable effect on both spinal levels.

### 3.8. Meta-Regression

**Figure 9:**
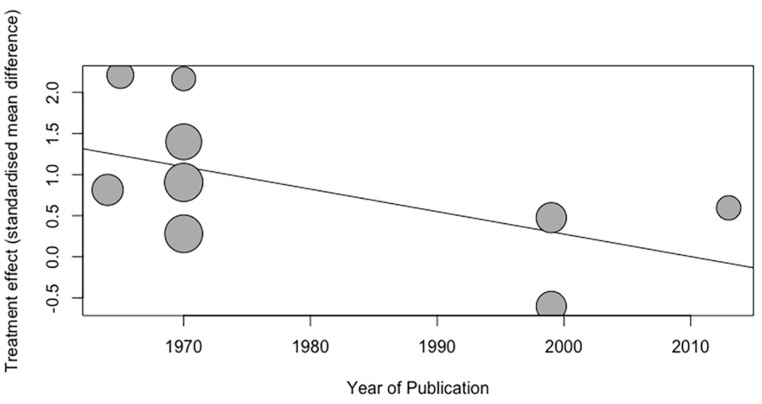
Meta-regression of the effect of posture according to the year of publication.

The meta-regression of publication year includes nine data points (Figure 9). The analysis indicated there was no significant correlation between the publication year and the estimated effect size associated with posture (Slope = −0.027, 95%CI= [−0.057, 0.002], *p* = 0.068). Meta-regression using the sample size as an independent variable showed that the sample size did not have a significant effect on effect size estimates (slope = 0.049, *p* = 0.875).

## 4. Discussion

The overarching goal of this review is to contrast the in vivo lumbar spine IDP measures between sitting and standing. The results of this review highlighted that sitting induces higher loads on the lumbar spine than standing in general. However, a meta-analysis of the more recent studies showed no significant difference between sitting and standing, which is consistent with the conclusion of the two reviews [21,22]. Besides, the trial sequential analysis showed that the overall effect size still falls within the adjusted monitoring boundary, indicating that more study is required. In addition, the degenerated discs showed no difference in IDP in both postures. 

### 4.1. Effect of Posture Variety in IDP Measurement

The meta-analysis of eligible studies showed that sitting induces a higher load on the lumbar spine than standing, which is consistent with earlier recommendations to avoid long sitting times in daily life [39]. However, most results were based on data from more than 30 years ago. In 2001, Wilke et al. [35] found that sitting and standing have similar effects on the lumbar spine IDP. They used a new implant transducer, a smaller apparatus, and their findings highly agreed with anthropometric data in many finite models. This improved equipment could stay securely in the IVD, thus ensuring that the measure is highly accurate and reliable. Rohlmann et al. [36] used internal spinal fixators and reported similar results in 1999. The implant of the vertebral body could restore normal load-bearing in the spine and collect the three degree-of-freedom force and moment data. Their results revealed that there is a higher load on the lumbar spine in standing because the upright position increases axial loads. The increase in lumbar lordosis in standing also raises the concave-sided compression force. These findings indicated that the improvement in measurement technique may lead to a dramatic difference in the observed in vivo IDP. 

The subgroup analysis that separated studies before and after the 1990s showed that there is no difference between the sitting and standing postures in more recent studies, which agrees with our hypothesis. However, only three studies [30,34,36] conducted in vivo IDP measurements after the 1990s and only 21 participants were involved. Therefore, these results should be interpreted with caution.

### 4.2. Effect of Disc Conditions and Levels on IDP Measurement

Because the measurements are of the pressure of the nucleus pulposus [40], according to previous studies, degenerative changes may affect the measure outcomes [41,42]. The bulging lamellae are squeezed by compressive load, and a disrupted disc partially loses the function of weight-bearing, thus showing higher stress in the annulus while reducing the nucleus pressure [43]. Considering the potential effects of pathological conditions in the disc, we performed a subgroup meta-analysis of the normal and the degenerated discs. Possibly, there is no difference of IDP between sitting and standing, and the degenerative changes do not change the comparison result, as both demonstrated the decreased measured value in general. Referring to the differences between disc levels, the results show no significant change in the effect size estimation, and indicated that the effect of posture could be similar on the two spinal segments. 

### 4.3. Large Variation in Outcomes

The variability of the IDP measure is high, even in similar disc conditions and in the same study, possibly because of the various types of transducers used. The earliest measurement [32] used a polyethylene-tipped liquid-filled transducer and obtained data nearly twice that of later measurements [19,34], 11 atmospheres compared with 5~6 atmospheres. Measurements using a piezoresistive needle and implanted transducers obtained similar outcomes, although confounding factors such as muscle activation and ligament responses still existed. Another possible source of error is the measurement process. The sequence of sitting and standing changes the body height, and prolonged standing reduces the disc height, thus lowering the spine loads [20] and eventually impacting the results. 

Moreover, considering individual factors, people with a higher weight will put more upper limb pressure on the same disc. Females with small nucleus pulposus areas along the spine will possibly have relatively lower IDPs. Thus, the variability of the IDP can be high if the participants have diverse body builds.

There is also some variation in the VBR measurement [29,30,31,36]. The location of each patient’s surgery level is varied, resulting in different outcomes. Moreover, in the early stages after surgery, patients may suffer from pain and psychological factors that restrict motion; therefore, the outcome difference may appear on the left and right sides and existing regional variations sometimes. Considering the implants in different spine levels and surgery setups within patients, sintered cancellous bone and bridged intact disc induce relatively high loads, while slight compression for implant mounting shows relatively lower loads [44].

### 4.4. Clinical Implication

Management of LBP includes several aspects. In addition to medicine, surgery, and psychological counselling to relieve pain [45,46], posture control is essential and preventative. Since Nachemson [33] reported that sitting led to a more significant loading on lumbar discs than standing, it was widely accepted that sitting poses more risks to intervertebral discs. The current review reveals that the previous viewpoint may not be correct, given the inconsistent findings of the more recent studies. Knowledge regarding the lumbar spinal loads in daily life is essential in LBP management [47]. Well recognized factors such as flexion and lifting weights result in a high spinal load [21,30,48]. The ‘postural perturbations’ strategy proposed recently can induce a high IDP, which interacts with the degenerated disc [49]. Existing conclusions are still controversial regarding the effect of the sitting and standing postures on IDP. Regardless of which posture induces a higher IDP, any prolonged posture is not recommended [50].

### 4.5. Limitation

This review has some limitations. First, the studies that undertook in vivo measurements are relatively few, and some of them were published decades ago and thus cannot provide the most recent data in this field and should be interpreted cautiously. Second, it is possible that there are duplicate subjects in some articles published by the same authors.

## 5. Conclusions

In conclusion, sitting induces higher loads on the lumbar spine than standing. However, this finding should be interpreted cautiously, as more recent studies indicate similar values or contradictory conclusions in both postures, even if they are in small quantities. The trial sequential analysis also indicates that the current number of studies may not provide sufficient statistical power. Furthermore, degenerated discs have a smaller IDP, and they show no difference in IDP in the sitting and standing postures may possibly result from the evenly distributed structures being ruptured. Furthermore, to maintain the well-functioning of the lumbar spine and manage LBP symptoms, any prolonged posture should be prohibited.

## Figures and Tables

**Table 1:** Characteristics of the included studies.

Study	Spinal Level	Sample Size Sitting	Sample Size Standing	Low Back Pain	Disc Condition	Transducer Type
Nachemson and Morris et al. 1964 [33]	L3-4	12	4	Yes	Normal	Liquid-filled
	L4-5	6	2	Yes	Normal	Liquid-filled
Nachemson et al. 1965 [32]	L3-4	10	10	Yes	Normal	Liquid-filled
Okushima et al. 1970 [25]	L3-4	10	10	Yes	Normal	Liquid-filled
	L4-5	20	20	Yes	Normal	Liquid-filled
	L3-4	7	7	Yes	Mid-degenerate	Liquid-filled
	L4-5	17	17	Yes	Mid-degenerate	Liquid-filled
	L3-4	6	6	Yes	Highly degenerate	Liquid-filled
	L4-5	17	17	Yes	Highly degenerate	Liquid-filled
Nachemson and Elfström et al. 1970 [37]	L3-4	7	9	No	Normal	Piezoresistive
Sato et al. 1999 [34]	L4-5	8	8	No	Normal	Piezoresistive, side-window
Rohlmann et al. 1999 [36]	between T12 and L5 levels	8	8	Not clear	Before anterior interbody fusion	Telemeterised internal spinal fixation devices
	between T12 and L5 levels	9	9	Not clear	After anterior interbody fusion	
Wilke et al. 2001 [35]	L4-5	1	1	No	Normal	implanted transducer
Rohlmann et al. 2008 [38]	between T12 and L3 levels	3	3	Not clear	vertebral fractures	Telemeterised vertebral body implant loading
Dreischarf et al. 2010 [31]	between T12 and L5 levels	5	5	Not clear	vertebral fractures	Telemeterised vertebral body implant loading
Rohlmann et al. 2013 [30]	Measured between T12 and L5 levels	5	5	Not clear	vertebral fractures	Telemeterised vertebral body implant loading

**Table 2:** Quality assessment of the included studies.

Study	Representativeness of the Study	Blinding of Outcome Assessment	Selective Reporting	Incomplete Outcome Data	Measurement Methods	Total
Nachemson 1964	*	-	*	*	*	4
Nachemson 1965	*	-	*	*	*	4
Okushima 1970	*	-	*	*	*	4
Nachemson 1970	*	-	*	*	*	4
Rohlmann 1999	*	-	*	*	*	4
Sato 1999	*	-	*	-	*	3
Wilke 2001	-	-	*	*	*	3
Rohlmann 2008	*	-	*	*	*	4
Dreischarf 2010	*	-	*	*	*	4
Rohlmann 2013	*	-	*	*	*	4

## Data Availability

No new data were created or analyzed in this study. Data sharing is not applicable to this article.

## Appendix A. Search Strategy

PubMed (to November 2021).

((“healthies”[All Fields] OR “healthy”[All Fields]) AND (“adult”[MeSH Terms] OR “adult”[All Fields] OR “adults”[All Fields] OR “adult s”[All Fields]) AND ((“sitting position”[MeSH Terms] OR (“sitting”[All Fields] AND “position”[All Fields]) OR “sitting position”[All Fields] OR “sitting”[All Fields] OR “sittings”[All Fields]) AND (“postural”[All Fields] OR “posturally”[All Fields] OR “posture”[MeSH Terms] OR “posture”[All Fields] OR “postures”[All Fields] OR “postured”[All Fields] OR “posturing”[All Fields])) AND ((“in vivo”[Journal] OR “in vivo brooklyn”[Journal] OR (“in”[All Fields] AND “vivo”[All Fields]) OR “in vivo”[All Fields]) AND (“spinal”[All Fields] OR “spinalization”[All Fields] OR “spinalized”[All Fields] OR “spinally”[All Fields] OR “spinals”[All Fields]) AND (“loaded”[All Fields] OR “loading”[All Fields] OR “loadings”[All Fields] OR “loads”[All Fields]))) OR ((“in vivo”[Journal] OR “in vivo brooklyn”[Journal] OR (“in”[All Fields] AND “vivo”[All Fields]) OR “in vivo”[All Fields]) AND (“intradiscal”[All Fields] OR “intradiscally”[All Fields]) AND (“pressure”[MeSH Terms] OR “pressure”[All Fields] OR “pressures”[All Fields] OR “pressure s”[All Fields] OR “pressurisation”[All Fields] OR “pressurised”[All Fields] OR “pressuriser”[All Fields] OR “pressurization”[All Fields] OR “pressurizations”[All Fields] OR “pressurize”[All Fields] OR “pressurized”[All Fields] OR “pressurizer”[All Fields] OR “pressurizes”[All Fields] OR “pressurizing”[All Fields])).

**Table A1:** Search strategy in Cochrane Database (to November 2021).

#	Searches
1	TITLE-ABS-KEY (spine load AND vivo)
2	TITLE-ABS-KEY (spine load AND vivo AND lumbar)
3	TITLE-ABS-KEY (lumbar AND spine AND load AND measurement)
4	TITLE-ABS-KEY (intradiscal pressure AND vivo)
5	TITLE-ABS-KEY (spine load AND sitting)
6	TITLE-ABS-KEY (spine load AND standing)
7	TITLE-ABS-KEY (spine load AND posture)
8	TITLE-ABS-KEY (lumbar intradiscal AND pressure)
9	TITLE-ABS-KEY (intradiscal pressure AND measure)
10	TITLE-ABS-KEY (Intervertebral disc AND pressure)
11	TITLE-ABS-KEY (Vertebral body replacement)

**Table A2:** Search strategy in Embase (to November 2021).

#	Searches
1	intradiscal pressure.mp.
2	Adult/
3	Posture/
4	sitting posture.mp.
5	standing posture.mp.
6	spinal loads.mp.
7	in vivo.mp.
8	Intervertebral Disc/
9	Humans/
10	vertebral body replacement.mp.
11	1 and 2 and 3
12	7 and 11
13	10 or 12
14	13 and 8

**Table A3:** Search terms in Google scholar (1940 to 2021) Any type of article.

#	Searches
1	Intradiscal pressure
2	Vertebral body replacement
3	Intervertebral disc pressure

**Table A4:** Search strategy in Scopus (to November 2021) Any type of article.

#	Searches
1	TITLE-ABS-KEY (intradiscal AND pressure)
2	TITLE-ABS-KEY (healthy AND adults)
3	TITLE-ABS-KEY (discal AND pressure)
4	TITLE-ABS-KEY (sitting AND posture)
5	TITLE-ABS-KEY (standing AND posture)
6	TITLE-ABS-KEY (vertebral AND body AND replacement)
7	TITLE-ABS-KEY (spinal AND loads)
8	(TITLE-ABS-KEY (spinal AND loads)) OR (TITLE-ABS-KEY (intradiscal AND pressure))
9	#8 AND #5
10	#8 AND #4
11	#8 AND #2
12	#8 AND #6
13	#6 AND #8 AND #4
14	#6 AND #8 AND #5
15	ALL (in vivo AND intradiscal AND pressure)
16	ALL (in vivo AND spinal AND loads OR intradiscal AND pressure)
17	ALL (vertebral AND body AND replacement AND intradiscal AND pressure AND adults AND lumbar AND spine)

**Table A5:** Search strategy in Web of Science (to November 2021).

#	Searches
1	ALL = (intradiscal pressure)
2	ALL = (spinal loads)
3	ALL = (sitting posture)
4	ALL = (standing posture)
5	ALL = (in vivo)
6	ALL = (healthy adults)
7	ALL = (vertebral body replacement)
8	ALL = (discal pressure)
9	ALL = (intradiscal pressure)
10	#1 AND #3
11	#1 AND #4
12	#1 OR #2
13	#12 AND #3
14	#12 AND #4
15	#5 AND #12
16	AB = (intradiscal pressure)
17	TI = (intradiscal pressure)
18	TI = (vertebral body replacement)
19	AB = (vertebral body replacement)
20	AB = (sitting)
21	AB = (lumbar spinal loads)
22	AB = (standing)
23	#21 AND #22
24	#21 AND #20
25	TI = (lumbar spinal loads)
26	TI = (intervertebral disc pressure)
27	TI = (disc pressure measurement)
28	ALL = (lumbar spine AND posture AND disc pressure)
29	ALL = (intradiscal pressure AND lumbar spine AND posture)

## Appendix B. List of Excluded Studies and Reasons for Exclusion

**Table A6:** List of Excluded Studies and Reasons for Exclusion.

Study	Reason for Exclusion				
	1	2	3	4	5
Nachemson, Morris et al. [51]	X				
Rohlmann, Graichen et al. [38]		X			
Rohlmann, Petersen et al. [52]		X			
Nachemson, [53]			X		
Zander and Dreischarf et al. [54]				X	
Rohlmann, Hinz et al. [55]				X	
Takahashi, Kikuchi et al. [56]				X	
Anderson, Oretengren et al. [57]				X	
Anderson, Oretengren et al. [58]				X	
Anderson, Oretengren et al. [59]					X
Anderson, Oretengren et al. [60]					X

Reasons:

1. Sample size of the study was not available.
2. Measurement performed on same subjects across studies.
3. Measurement performed on cadaver subject.
4. Results did not contain both sitting and standing measurement data.
5. Results were presented in graphs only, and the numerical data could not be extracted.
